# Reduced Anisotropic in Thermal Conductivity of Polymer Composites via Chemically Bonded BN–SiC Hybrid Fillers

**DOI:** 10.3390/polym17192580

**Published:** 2025-09-24

**Authors:** Won-Jin Kim, Mi-Ri An, Sung-Hoon Park

**Affiliations:** Department of Mechanical Engineering, Soongsil University, 369 Sangdo-ro, Dongjak-gu, Seoul 06978, Republic of Korea; dnjswlso@naver.com (W.-J.K.); anmiri0622@naver.com (M.-R.A.)

**Keywords:** thermal interface materials (TIM), boron nitride, silicon carbide, polymer composite, anisotropic ratio

## Abstract

The growing demand for efficient thermal management in power electronics and high-density optoelectronic systems necessitates thermal interface materials (TIMs) with high through-plane thermal conductivity and minimal anisotropy. However, conventional polymer composites filled with platelet-type fillers such as hexagonal boron nitride (h-BN) suffer from strong directional thermal transport and interfacial resistance, limiting their practical effectiveness. To address this limitation, we present a hybrid filler strategy wherein h-BN and silicon carbide (SiC) nanoparticles interact via hydroxylated surfaces, forming a three-dimensional thermally conductive network. The resulting BN–SiC composite exhibits enhanced through-plane thermal conductivity (1.61 W/mK at 70 vol%) and lower anisotropy ratios (<2.0 at 30 vol%), all while maintaining mechanical integrity and processability. These results demonstrate that chemical bonding at the filler interface can reduce interfacial thermal resistance and extend thermal conduction paths three-dimensionally, providing insights into interface-based heat transfer mechanisms. This strategy presents a scalable and practical approach for next-generation thermal management solutions in electronic packaging and high-power device platforms.

## 1. Introduction

Effective thermal management has become increasingly critical in power-intensive applications such as power semiconductors, high-brightness LEDs, electric vehicles, and aerospace electronics where rising integration density and escalating heat generation compromise system stability, efficiency, and reliability [1,2,3,4]. Among the various thermal control strategies, thermal interface materials (TIMs) serve as vital interstitial layers that reduce interfacial thermal resistance between heat sources and heat sinks [5,6,7]. Polymer-based TIMs are particularly attractive owing to their conformability, electrical insulation, and ease of large-area processing. However, their intrinsically low thermal conductivity (typically 0.1–0.3 W/mK for pristine polymers) necessitates the incorporation of high-performance thermally conductive fillers to meet next-generation thermal demands [8,9,10,11].

A variety of thermally conductive fillers such as aluminum oxide (Al_2_O_3_), aluminum nitride (AlN), silicon carbide (SiC), and graphene have been studied to improve the thermal performance of polymer-based TIMs [12,13,14]. Al_2_O_3_ and AlN offer good thermal conductivity with electrical insulation but often require high loadings to reach sufficient conductivity levels, which can compromise mechanical integrity and processability. Graphene and carbon nanotubes exhibit outstanding intrinsic thermal conductivity [15,16]; however, their electrical conductivity limits their use in insulating applications, and their tendency to agglomerate poses significant challenges to dispersion [17,18,19]. SiC, while offering balanced thermal and mechanical properties, cannot typically form percolated networks due to its particulate nature unless surface modifications or structural design strategies are employed [20]. These limitations highlight the need for advanced filler architectures that not only combine favorable properties from multiple materials but also promote efficient phonon transport across interfaces and in all spatial directions.

Hexagonal boron nitride (h-BN) has attracted attention as a promising filler due to its high intrinsic in-plane thermal conductivity (reported to be up to ~750 W/mK), electrical insulation, and chemical inertness [21,22,23]. Nonetheless, its strong platelet anisotropy leads to preferential in-plane alignment during processing, resulting in poor through-plane thermal conduction and severe transport imbalance [24]. Moreover, weak interfacial interactions between fillers hinder phonon continuity, preventing the formation of efficient, isotropic thermal pathways. To overcome these intrinsic limitations, numerous strategies have been explored including vertical alignment of BN [25,26,27], surface functionalization of BN [28,29,30], physical hybrid system of BN and other fillers [31,32,33], and covalent bond between BN and other fillers [34,35]. However, recent studies still have limitations. For example, hybrid systems that simply physically combine BN and various nanoparticles have shown some improvement in thermal conductivity; however, because they primarily rely on direct contact between particles, they may not completely resolve the thermal resistance and heat transfer discontinuity issues that occur at the interface. On the other hand, studies introducing covalent bonds between BN and nanoparticles can effectively reduce thermal resistance at the interface, but such methods require complex processes or may not sufficiently consider the directionality of heat conduction within the composite. In other words, although previous studies have contributed to improving thermal conductivity, a practical and scalable design strategy is needed that can achieve excellent thermal conductivity not only in the planar direction but also in the thickness direction while minimizing anisotropy.

Thus, in this work, we propose a chemically engineered hybrid filler system comprising base-treated h-BN and acid-functionalized silicon carbide (SiC) nanoparticles, which are covalently bonded via a condensation reaction under basic conditions. SiC was selected for its spherical morphology, high intrinsic thermal conductivity (30~270 W/mK for β-SiC) [36], and surface reactivity after acid treatment, which enables not only covalent linkage to BN but also hydrogen bonding among adjacent SiC particles. This dual-interaction mechanism ensures both vertical thermal bridging across BN platelets and suppression of interfacial thermal resistance within the filler domain [37,38]. When uniformly dispersed into a PDMS matrix via high-shear three-roll milling, the BN–SiC hybrid forms a robust, percolated three-dimensional thermal network. The resulting composite demonstrates significantly enhanced through-plane thermal conductivity and a reduced anisotropic ratio compared to conventional BN-based systems. Moreover, infrared thermography conducted under device-relevant conditions confirms the material’s ability to dissipate heat rapidly and uniformly, validating its performance at the application level. By integrating chemically bonded BN and SiC within a PDMS matrix, this study establishes a scalable design strategy for polymer composites that simultaneously enhance through-plane thermal transport and suppress anisotropy, thereby overcoming the intrinsic limitations of conventional BN-based TIMs.

## 2. Materials and Methods

### 2.1. Materials

Hexagonal boron nitride (APS: 6–8.5 μm, 3M, Saint Paul, MN, USA) was used as the primary ceramic filler. Silicon carbide (APS: 45–65 nm, RNDKOREA, Seoul, Republic of Korea) nanoparticles were employed as a secondary filler for hybridization. Sodium hydroxide beads (Sigma-Aldrich, St. Louis, MO, USA) and aqueous ammonia solution (37%, Sigma-Aldrich, St. Louis, MO, USA) were used for surface functionalization of h-BN and as a base for condensation reactions, respectively. Ethanol (Samchun Chemicals, Seoul, Korea) and concentrated nitric acid (70%, Samchun Chemicals, Seoul, Republic of Korea) served as solvents and acid treatment agents. Polydimethylsiloxane (Sylgard 184, Dow Corning, Midland, MI, USA) was adopted as the polymer matrix. Concentrated sulfuric acid (95%, Daejung Chemicals, Siheung, Republic of Korea) was used for acid treatment of SiC. All chemicals were used as received without further purification.

### 2.2. Synthesis of BN–SiC Hybrid Filler

The BN–SiC hybrid filler was synthesized through a multi-step process comprising surface functionalization of hexagonal boron nitride (h-BN) and silicon carbide (SiC), followed by a condensation reaction between the two components.

First, h-BN powder was functionalized by alkaline treatment to introduce hydroxyl groups onto its surface. Specifically, 1 L of deionized water was prepared, and sodium hydroxide (NaOH) was added to achieve a 5 M concentration. 10 g of h-BN was dispersed in this solution and stirred at 120 °C for 24 h at 200 rpm. After the reaction, the product was collected by vacuum filtration, thoroughly washed with deionized water, and dried in an oven at 100 °C for 24 h to yield hydroxyl-functionalized BN (OH–BN).

In parallel, SiC nanoparticles were functionalized by acid treatment. A mixed acid solution was prepared by combining 75 mL of concentrated sulfuric acid (95%) with 25 mL of concentrated nitric acid (70%). SiC powder (4 g) was added to this solution and stirred at 60 °C for 3 h at 200 rpm. After the reaction, the mixture was diluted with an excess of deionized water, filtered, and dried in an oven at 100 °C for 24 h to produce hydroxyl-functionalized SiC (OH–SiC).

For the hybridization step, OH–BN and OH–SiC were mixed at a 2:1 mass ratio. Both components were dispersed in 160 mL of anhydrous ethanol, and aqueous ammonia was added until the pH reached approximately 11. The suspension was stirred at 60 °C for 12 h at 200 rpm to induce chemical reactions between the hydroxyl groups present on the surfaces of BN and SiC. After the reaction, the product was diluted with ethanol, collected by filtration, and dried at 80 °C for 24 h to yield the final BN–SiC hybrid filler.

### 2.3. Fabrication of Polymer Composites with BN–SiC Hybrid Filler

**Figure 1:**
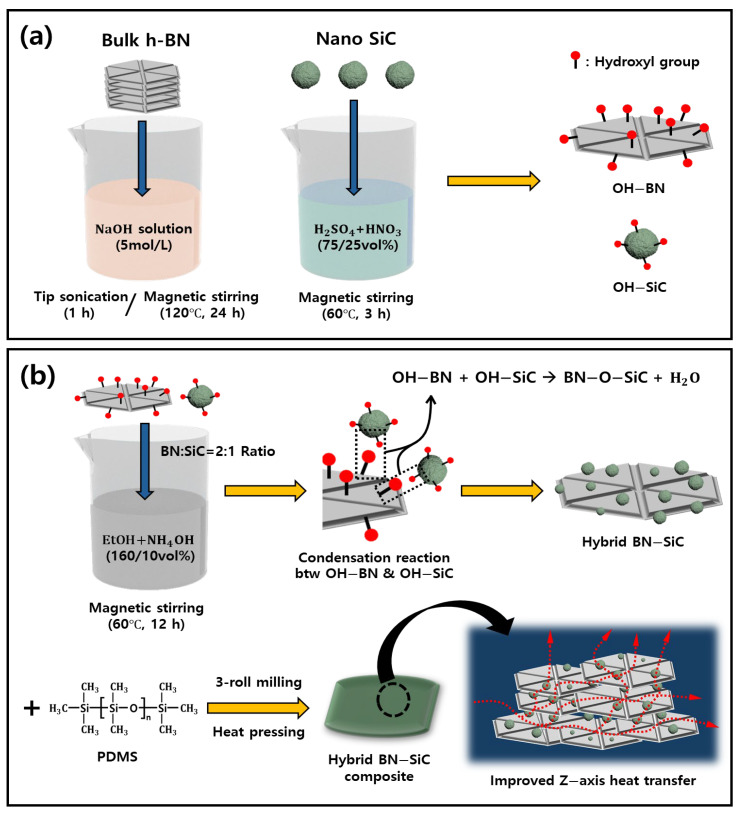
Schematic illustration of the synthesis and integration process of the BN–SiC hybrid filler into a polymer composite: (**a**) Surface functionalization of micron-sized hexagonal boron nitride (h-BN) and nano-sized silicon carbide (SiC) through alkaline and acid treatments, respectively, to introduce hydroxyl groups. (**b**) Bonding of OH–functionalized BN and SiC via chemical reaction in a basic ethanol solution (BN:SiC = 2:1 by weight) followed by dispersion into a PDMS matrix using three-roll milling for homogeneous distribution, and thermal curing to obtain the final composite.

For each formulation, 4 g of PDMS resin was used with a curing agent at a weight ratio of 10:1 (PDMS: curing agent). The previously synthesized BN–SiC hybrid filler was then incorporated at volume fractions of 30 vol%, 50 vol%, and 70 vol%, depending on the target composition. The PDMS resin, curing agent, and hybrid filler were initially blended using a paste mixer to achieve preliminary dispersion. To further enhance filler distribution and reduce agglomeration, the mixture was processed using a three-roll milling apparatus for 90 s. The resulting composite paste was then transferred into a mold with a thickness of 200 μm, and the paste was repeatedly tapped on a hard surface after being placed on a press plate to remove trapped air bubbles. The mold was subjected to thermal curing at 150 °C for 1 h, resulting in a solid composite in which the BN–SiC hybrid filler was uniformly embedded within the PDMS matrix. For comparison, control composites were also prepared using either pristine BN alone or a physical mixture of BN and SiC (mass ratio of 2:1). These reference samples were fabricated using the same processing steps and filler volume fractions (30, 50, and 70 vol%) as the hybrid-filled composites. The overall fabrication process of the BN–SiC hybrid filler and its integration into the PDMS is illustrated in Figure 1.

### 2.4. Characterization

To examine the dispersion state of the BN–SiC hybrid filler and its interfacial adhesion within the PDMS matrix, the cross-sectional morphology of the composite was observed using scanning electron microscopy (SEM, Gemini SEM 300, Zeiss, Oberkochen, Land Baden-Württemberg, Germany). SEM imaging was performed at an accelerating voltage of 3 kV, and the specimens were cryofractured in liquid nitrogen to preserve their native internal microstructure. To confirm the chemical bonding between the surface-treated fillers, Fourier-transform infrared spectroscopy (FT-IR, Spectrum 2000, Perkin Elmer, Waltham, MA, USA) was employed in transmission mode. Each sample was pelletized with KBr, and spectra were acquired over the range of 4000–500 cm^−1^. Thermal conductivity was measured using a transient plane source method with a TPS 2500S instrument (Hot Disk, Gothenburg, Sweden). The measurements were performed according to the ISO 22007-2 standard [39] using a 5465 sensor [40]. Both in-plane (using a slab module) and through-plane (using a thin film module) thermal conductivities were measured using composite samples manufactured in the shape of a disk (diameter: 3 cm, thickness: 200 μm). All measurements were performed five times, and the average value was calculated.

For thermal dissipation evaluation, a custom-built LED test platform was used. The TIM (Pure PDMS & BN–SiC 70 vol% hybrid composite, cut into 3 × 3 cm pieces) was inserted between the LED and the heat sink, with a thin layer of commercial thermal grease (~3 W/mK, Thermal-Grizzly, Brandenburg, Germany) applied at both interfaces to minimize contact resistance. Then, a DC power supply (EX300-50, ODA Technology, Incheon, Korea) was connected to a 2 × 2 cm LED chip (GREATZT, Shanghai, China) to create a constant heat source. During testing, the ambient temperature was maintained at room temperature (20 °C). Surface temperature profiles were monitored over time using an infrared thermal imaging camera (M11W, NAVIMRO, Seoul, Republic of Korea) during continuous power operation.

## 3. Results and Discussion

### 3.1. Morphology Analysis

**Figure 2:**
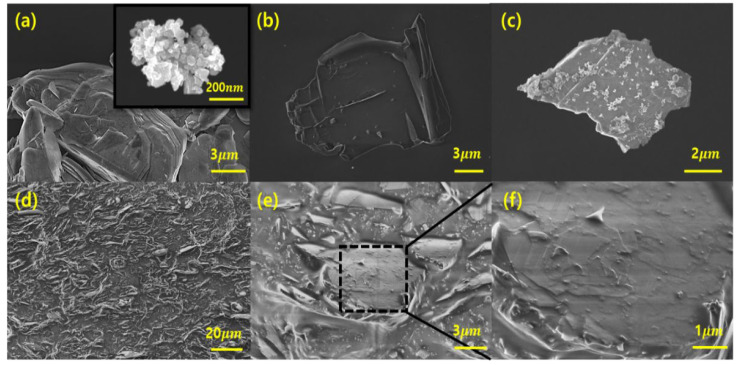
SEM images of (**a**) Bulk micro h-BN (inset: Bulk nano SiC), (**b**) OH–BN, (**c**) condensation-reacted BN–SiC hybrid filler (2:1 ratio), (**d**–**f**) Cross-sectional SEM images at low (**d**), medium (**e**), and high (**f**) magnifications of a PDMS/BN–SiC composite (70 vol%).

As shown in Figure 2, the morphological characteristics of the base material and hybrid filler were investigated using scanning electron microscopy (SEM). Figure 2a shows hexagonal boron nitride (h-BN) powders in the form of flakes with a lateral size of approximately 5–10 μm, which are composed of a platelet structure with a layered structure [41]. This morphology is favorable for lateral phonon transport but fundamentally limits the through-plane (Z-axis) thermal conductivity due to the two-dimensional layered structure and insufficient vertical connectivity. The inset image shows fine silicon carbide (SiC) nanoparticles with a quasi-spherical shape of less than 100 nm, which are ideal for hybridization with micro-sized BN.

Figure 2b shows BN flakes (OH–BN) surface-modified with a strong base (NaOH), a decrease in both the size and thickness of the BN particles due to the combined effects of sonication and alkaline stirring [42]. This process increases the specific surface area, which facilitates effective interaction with SiC nanoparticles and provides more anchoring sites for subsequent reactions. After reaction under alkaline conditions (pH 10–11), the resulting BN–SiC hybrid filler exhibits SiC nanoparticles uniformly anchored on the surface and edges of BN flakes (Figure 2c). The SiC nanoparticles are homogeneously distributed on the BN surface without serious agglomeration, indicating successful hybridization between the two components. This engineered interface formed through chemical bonds is fundamentally different from a simple physical mixture or blend. The SiC particles serve as vertical links between the in-plane oriented BN flakes during the process, contributing to reducing the interfacial thermal resistance. As a result, the spatially continuous and chemically bonded network forms the basis for three-dimensional phonon transport, which is a key design principle for enhancing the interfacial thermal conductivity.

Cross-sectional SEM images of PDMS composites containing 70 vol% BN–SiC filler are shown in Figure 2d–f at increased magnification. Figure 2d,f demonstrate the uniform dispersion of the fillers throughout the matrix and close interfacial contact without voids. Especially, the high magnification image in Figure 2f demonstrates that the BN and SiC components of the hybrid filler are tightly interconnected even after intensive shear mixing via 3-roll milling. This microstructural configuration plays a pivotal role in governing thermal performance. Unlike the mixed BN and SiC fillers, which often suffer from interfacial gaps and random dispersion, the BN–SiC structure forms a vertically continuous thermal path, reducing the interfacial thermal resistance and overcoming the inherent anisotropy of BN-based composites.

### 3.2. Confirmation of Chemical Bonding Via FT-IR Analysis

**Figure 3:**
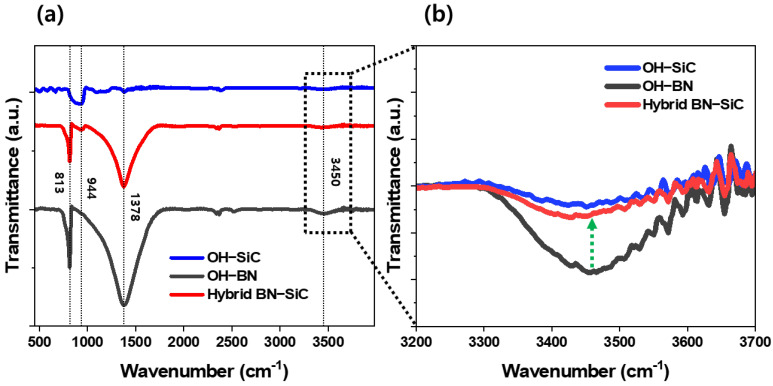
FT-IR spectra of (**a**) OH–BN, OH–SiC, and BN–SiC hybrid filler in the full range (500–4000 cm^−1^), and (**b**) magnified view of the –OH stretching region (3200–3700 cm^−1^) showing reduction in hybrid BN–SiC’s hydroxyl peak.(Green arrow: indicating the reduction of –OH stretching intensity in the hybrid BN–SiC).

To validate the formation of chemical bonds between the hydroxyl-functionalized fillers, Fourier-transform infrared (FT-IR) spectroscopy was conducted on the base-treated h-BN (OH–BN), acid-treated SiC (OH–SiC), and the BN–SiC hybrid filler. The resulting spectra are presented in Figure 3.

As shown in Figure 3a, OH–BN exhibits prominent peaks near 1378 cm^−1^ and 813 cm^−1^, which correspond to the in-plane stretching and out-of-plane bending vibrations of B–N bonds, respectively [43,44]. These characteristic bands confirm that the hexagonal BN structure remains intact after base treatment, preserving its high intrinsic thermal conductivity. Plus, OH–SiC shows a distinct absorption band at approximately 700–1000 cm^−1^, attributable to the Si–C bond stretching [45], indicating that the SiC crystal framework also remains stable following acid treatment. More importantly, the FT-IR spectrum of the BN–SiC hybrid filler shows all key signatures of both precursors with slightly reduced peak intensities, suggesting the preservation of the parent structures after condensation. The hybridization process does not disrupt the underlying chemical framework of either component, which is an essential factor for maintaining the inherent thermal performance of the individual fillers.

The most critical insight is provided in the high-wavenumber region (3200–3700 cm^−1^), as shown in Figure 3b, where broad O–H stretching bands are observed in both OH–BN and OH–SiC samples [45,46]. These peaks originate from hydroxyl groups introduced during surface modification and serve as essential precursors for the subsequent reaction. Notably, in the BN–SiC spectrum, the intensity of the O–H stretching band significantly diminishes, indicating substantial consumption of surface hydroxyls during condensation and suggesting the possible formation of covalent linkages between the hydroxylated precursors at the interface [47,48].

Although FT-IR alone does not unequivocally prove covalent bond formation, the pronounced reduction in hydroxyl vibrations, when correlated with the enhanced thermal transport performance, provides strong indirect evidence of interfacial chemical coupling. This interfacial bonding enhances the mechanical integrity and thermal continuity of the hybrid filler, which is reflected in the improved through-plane thermal conductivity and reduced anisotropy. Thus, the FT-IR results confirm that the BN–SiC hybrid structure is not a simple physical mixture but rather a chemically integrated architecture, designed to function as a 3D thermal bridge [38,49]. This chemical integration is central to the hybrid filler’s ability to mitigate interfacial thermal resistance and maximize heat transfer efficiency in polymer-based thermal interface materials.

### 3.3. Evaluation of Thermal Conductivity and Anisotropy

To evaluate the influence of hybrid filler composition on the thermal transport characteristics of the composite, a series of samples with a fixed total filler loading (70 vol%) were prepared by systematically varying the BN:SiC weight ratio from 1:1 to 6:1. This compositional design was motivated not by differences in filler identity alone, but rather by the structural and interfacial interplay arising from the combination of two distinct fillers—one platelet-shaped (BN) and one spherical (SiC)—with inherently different morphologies and surface chemistries. The goal was to investigate how their relative ratios affect the formation of multidirectional thermal pathways and to identify an optimal structural-functional balance. Table 1 presents the foundational dataset that supports the thermal conductivity and anisotropy trends.

As shown in Table 1, increasing the BN content monotonically enhances the in-plane (X-Y) thermal conductivity due to the lateral alignment of high-aspect-ratio BN platelets during processing. This alignment facilitates effective phonon transport along the planar direction. In contrast, through-plane (Z-axis) conductivity exhibits a non-monotonic trend, reaching a maximum value at a BN:SiC ratio of 2:1 and decreasing at both lower and higher BN contents.

This trend is driven not just by how much filler is added, but by how well the fillers are connected to each other and how effectively heat can cross their interfaces. SiC, owing to its spherical morphology and surface hydroxyl functionalities, can form hydrogen bonds with neighboring SiC particles, while also participating in covalent bonding with BN platelets. At the 2:1 ratio, the SiC content is sufficient to serve as thermally conductive vertical bridges between adjacent BN layers, promoting both through-plane continuity and interfacial coupling. At the 1:1 ratio, the higher SiC content might be expected to enhance vertical thermal transport; however, the excessive number of spherical particles interferes with the ordered arrangement of BN platelets. This disruption not only weakens the lateral thermal network but also prevents the SiC particles from forming a well-connected vertical bridge structure, as they become more randomly dispersed rather than selectively positioned between BN layers. Consequently, the X-Y and Z-axis thermal conductivity all at 1:1 remains lower than at 2:1, where the balance between lateral BN alignment and vertical SiC bridging is optimized. Accordingly, the 2:1 composition between BN and SiC represents the optimal balance point where both vertical and lateral thermal pathways are synergistically developed. For this reason, the 2:1 hybrid filler formulation was selected for all subsequent thermal performance analyses throughout this study.

**Figure 4:**
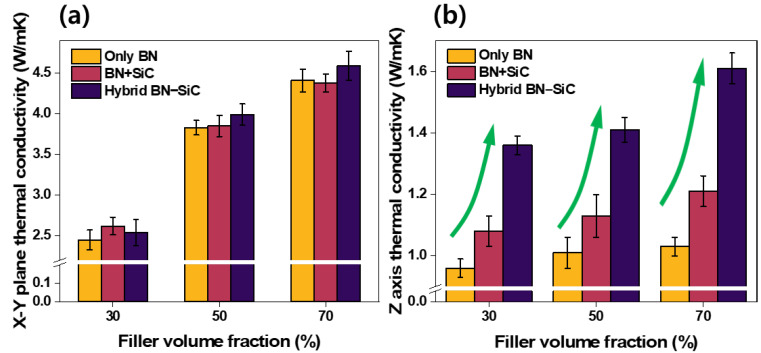
(**a**) In-plane (X-Y) thermal conductivity and (**b**) through-plane (Z-axis) thermal conductivity of composites using only BN, a simple physical mixture of BN and SiC (2:1 ratio), and the BN–SiC hybrid filler. Each filler was added at the indicated volume fractions.(Green arrows: highlighting the pronounced enhancement in Z-axis thermal conductivity achieved by using the BN–SiC hybrid filler).

The thermal conductivity performance of PDMS-based composites incorporating various filler systems was evaluated in both in-plane (X-Y) and through-plane (Z-axis) directions. As shown in Figure 4, three representative formulations were compared: (i) composites with h-BN as the sole filler, (ii) composites with a simple physical mixture of h-BN and SiC (2:1 mass ratio), and (iii) composites containing the chemically bonded BN–SiC hybrid filler. Each system was tested at filler loadings of 30, 50, and 70 vol%.

Figure 4a presents the in-plane thermal conductivity results. As expected, all BN-containing systems show relatively high in-plane values due to the strong phonon transport along the basal planes of BN platelets (~750 W/mK). Notably, there is minimal variation among the three systems at a given loading, indicating that in-plane heat transport is primarily governed by the intrinsic properties and alignment of BN flakes. The inclusion of SiC—either as a simple additive or through hybridization—does not significantly enhance or degrade in-plane performance. This outcome confirms that the design of the hybrid filler does not compromise the lateral thermal pathways established by the high aspect ratio of BN platelets.

In contrast, Figure 4b demonstrates a stark difference in through-plane thermal conductivity. At 30 vol% filler loading, the hybrid filler composite achieves a value of 1.36 W/mK, compared to 0.96 W/mK for the BN-only composite and 1.08 W/mK for the BN/SiC mixture. This trend persists at 50 and 70 vol%, where the BN–SiC composite reaches 1.61 W/mK at 70 vol%, outperforming both the BN-only (1.03 W/mK) and the physically mixed BN/SiC (1.21 W/mK) counterparts. The superior through-plane performance of the BN–SiC system is directly attributable to the morphology and chemistry of the hybrid filler. As revealed in Figure 2, the SiC nanoparticles are strategically positioned on the surfaces of BN flakes, acting as thermally conductive bridges between horizontally aligned platelets. This arrangement facilitates phonon transfer across the normally discontinuous inter-flake regions, which are major bottlenecks in conventional BN composites. Moreover, the chemical bonding confirmed in FT-IR analysis (Figure 3) minimizes interfacial thermal resistance by eliminating weak, phonon-scattering interfaces.

The incremental improvement from physical mixture (BN+SiC) to chemical hybrid (BN–SiC) underscores the significance of engineered interfacial connectivity. In the physically mixed system, SiC nanoparticles are randomly dispersed and poorly integrated with BN flakes, limiting their ability to form continuous thermal pathways. By contrast, the hybrid filler establishes a 3D percolation network with enhanced continuity along the Z-direction—a critical requirement for practical TIM applications where heat must be efficiently dissipated perpendicular to the interface. Collectively, these results demonstrate that the BN–SiC hybrid filler system effectively enhances through-plane thermal conductivity without compromising in-plane performance [50].

**Figure 5:**
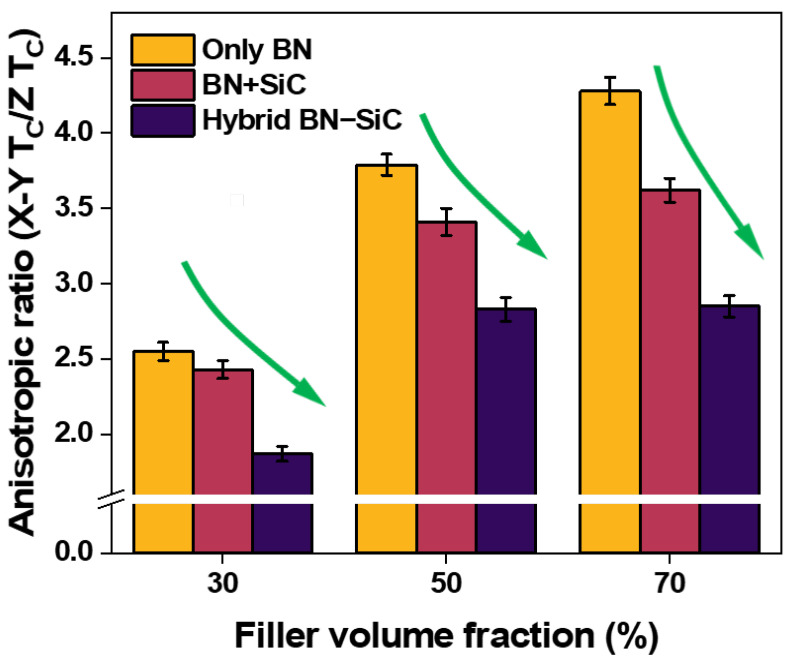
Anisotropic ratio (in-plane to through-plane thermal conductivity) of composites using only BN, a simple BN and SiC mixture (2:1 ratio), and the condensation-reacted BN–SiC hybrid filler.(Green arrows: highlighting the significant decrease of the anisotropy ratio achieved using BN–SiC hybrid fillers).

Thermal conductivity anisotropy is a critical limitation in polymer composites filled with 2D materials such as hexagonal boron nitride (h-BN). Due to their intrinsic platelet morphology, h-BN flakes tend to align in-plane during processing, resulting in highly directional heat flow and inefficient dissipation through the thickness. To quantify the anisotropy in our systems, the anisotropic ratio—defined as the ratio of in-plane to through-plane thermal conductivity—was calculated [51]. The results are presented in Figure 5.

At 30 vol% filler loading, the anisotropic ratio of the BN-only composite reaches 2.55, reflecting significant disparity between in-plane (2.45 W/mK) and through-plane (0.96 W/mK) conductivities. The BN+SiC mixture shows slight improvement (2.43), due to limited contributions from randomly dispersed SiC particles. However, BN–SiC hybrid filler markedly mitigates the anisotropic ratio to 1.87, thereby enabling more isotropic thermal transport. This trend is consistent until higher filler loading (70 vol%), where the anisotropic ratio of the hybrid system is lowered to 2.87 compared to 4.28 and 3.62 for the BN-only and BN+SiC systems, respectively. These results directly correlate with the 3D microstructural architecture observed in Figure 2 and the through-plane conductivity enhancement described in Figure 4b. The marked decrease in anisotropy confirms that SiC nanoparticles in the hybrid system effectively bridge adjacent BN platelets in the vertical direction, thus mitigating the directional bias inherent in 2D filler systems.

From a thermal interface material (TIM) design perspective, this isotropization of heat transport is especially valuable. In practical applications such as power electronics, LED packaging, and EV battery modules, thermal flux is not confined to a single direction. Therefore, composites that support multidirectional heat dissipation offer superior performance and reliability. The BN–SiC achieves this by translating filler-level design (chemical hybridization and spatial orientation) into macroscale isotropic transport behavior. Taken together, the data in Figure 4 and Figure 5 strongly support the central hypothesis of this work: that engineered hybridization of h-BN with SiC can overcome the fundamental limitations of platelet-dominated systems. By providing through-thickness connectivity while preserving high in-plane conductivity, the BN–SiC filler enables thermally isotropic composites that meet the stringent demands of modern thermal management applications.

### 3.4. Heat Transfer Mechanism

**Figure 6:**
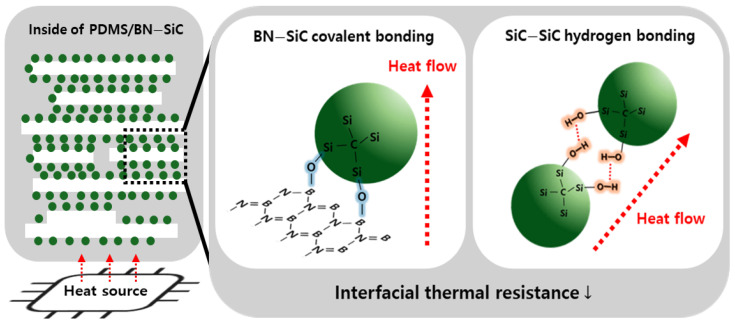
Schematic of interfacial heat transfer in PDMS/BN–SiC composites. (**Left**): SiC nanoparticles (green) form a 3D network bridging BN platelets (white) in the polymer matrix. (**Middle**): Covalent bonding at BN–SiC interfaces enhances thermal connectivity and heat flow. (**Right**): Hydrogen bonds between surface –OH groups at SiC–SiC interfaces further reduce interfacial thermal resistance, promoting phonon transfer.

To elucidate the structural origin of enhanced through-plane thermal conductivity in the BN–SiC composites, a conceptual schematic is provided in Figure 6. This model integrates morphological, chemical, and thermal findings presented in Figure 2, Figure 3, Figure 4 and Figure 5 and visualizes the multi-scale thermal conduction pathways established by the hybrid filler. As shown in the schematic, h-BNs tend to align parallel to the matrix during heat curing under high pressure, because of their intrinsic plate-like structure [52,53]. While this promotes high in-plane thermal conductivity, it leaves the through-plane direction poorly connected, with phonon transport disrupted by inter-flake voids and interfacial scattering. As a result, vertical heat dissipation is significantly hindered, and strong anisotropy emerges (Figure 5).

However, the BN–SiC hybrid filler chemically grafted onto BN surfaces fundamentally alters this configuration. First, SiC nanoparticles are chemically grafted onto the surface and edges of BN platelets Via chemical reactions between surface hydroxyl groups, which may form B–O–Si or Si–O–B linkages. These bridges provide robust vertical interconnectivity between neighboring BN platelets, effectively transforming the filler network from 2D into a quasi-3D percolated structure that supports phonon transport in the through-thickness direction. Second, the SiC nanoparticles themselves—bearing hydroxyl-rich surfaces due to acid treatment—form inter-particle hydrogen bonds (SiC–SiC). These hydrogen bonding interactions serve to reduce the thermal boundary resistance (TBR) between adjacent SiC nanoparticles [54,55], especially when they accumulate between parallel BN flakes. Although weaker than covalent bonds, hydrogen bonds help suppress interfacial phonon scattering at the nano-level across SiC–SiC interfaces without requiring additional binders or matrix mediation [56]. This dual mechanism can greatly enhance the thermal continuity and reduce phonon scattering across filler junctions [49,57,58,59].

In addition, the presence of SiC nanoparticles with relatively high intrinsic thermal conductivity enhances local thermal diffusion within the matrix and reinforces the mechanical stability of the composite. This dual function—thermal bridging and structural reinforcement—makes SiC an ideal partner for BN in hybrid filler design. The resulting hybrid network thus enables simultaneous exploitation of BN’s lateral transport and SiC’s vertical bridging capability. This comprehensive bridging strategy explains the marked improvements in through-plane thermal conductivity and the significant reduction in anisotropy (Figure 5).

### 3.5. Experimental Setup for Thermal Performance Evaluation

**Figure 7:**
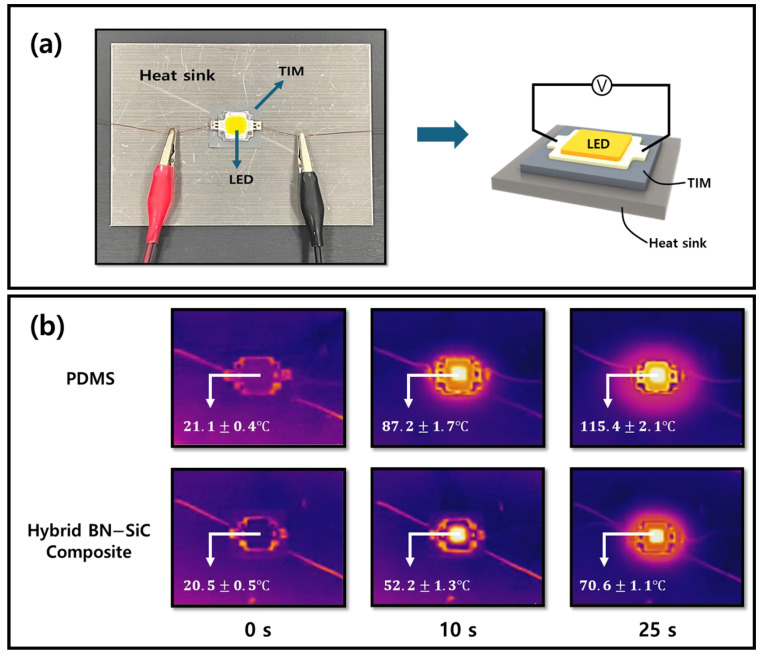
Custom-built platform for evaluating thermal conductivity performance of BN–SiC 70 vol% composite and PDMS under controlled heating conditions. (**a**) Schematic and photographic view of the experimental heat dissipation test platform, where an LED is supplied with 10 V voltage, with a thermal interface material (TIM)—either pure PDMS or the hybrid BN–SiC composite—applied between the LED and the heat sink. (**b**) Time-resolved infrared thermographic images capturing the surface temperature evolution of the LED after voltage application (0, 30, and 60 s). The top row presents results using PDMS as the TIM, and the bottom row displays results with the hybrid BN–SiC composite TIM.

To demonstrate the practical heat dissipation capabilities of the BN–SiC composite, real-time thermal diffusion was visualized using an infrared (IR) thermal imaging platform, as shown in Figure 7. The thermal interface material (TIM)—either pure PDMS or the BN–SiC composite (70 vol%)—was inserted between the LED and the heat sink, and surface temperatures were monitored via infrared (IR) thermography during continuous power supply at an applied voltage of 10 V, as illustrated in Figure 7a. The 10 V voltage is the maximum usable value, since voltages above 11 V caused local burning and smoke generation at the PDMS.

Time-resolved IR thermal images in Figure 7b reveal the striking contrast in heat dissipation between the two TIM systems. In the top row, when pure PDMS is used as the TIM, the LED surface temperature rapidly increases from 21.1 °C to 115.4 °C within 60 s, then gradually reaches saturation. This severe thermal accumulation originates from the inherently low thermal conductivity of PDMS (~0.1 W/mK), which limits effective heat spreading within the TIM layer and causes localized thermal build-up around the LED chip. In contrast, the bottom row shows the LED behavior with the BN–SiC hybrid composite as the TIM, where the temperature rises more moderately and saturates at ~70 °C after 60 s. Compared with PDMS, the BN–SiC composite significantly lowers the hottest spot temperature of the LED, highlighting its superior through-plane heat transfer capability.

The superior thermal response of the BN–SiC system is attributable to multiple factors, as established in prior sections. The hybrid filler forms a chemically bonded 3D percolated network (Figure 6), effectively minimizing thermal boundary resistance both at the BN–SiC interface and between SiC nanoparticles through hydrogen bonding. The chemically bonded 3D network enables phonons to travel efficiently in all directions, improving heat transfer across both the lateral plane and the composite thickness. In addition, the high filler loading (70 vol%) establishes a robust percolation network, further enhancing the overall thermal conductivity of the composite.

Collectively, the results in Figure 7 provide compelling evidence that the BN–SiC hybrid filler system enables thermally responsive, structurally stable composites suitable for demanding heat management applications, including power modules, LED packages, and high-frequency electronic components. The ability to rapidly dissipate localized heat with minimal anisotropy is particularly advantageous in preventing thermal hotspots and extending device longevity.

## 4. Conclusions

This study presents a rationally designed thermal interface material (TIM) that utilizes a chemically bonded BN–SiC hybrid filler to overcome the inherent limitations of conventional polymer composites, namely directional anisotropy and interfacial thermal resistance. The hybrid filler is formed through a chemical reaction between hydroxyl-functionalized hexagonal boron nitride (h-BN) and silicon carbide (SiC) nanoparticles, generating a three-dimensional percolated thermal network. As a result, at a high filler content of 70 vol%, the through-plane thermal conductivity of the composite reached 1.61 W/mK, and the anisotropy ratio was significantly reduced to 2.87 compared to 4.28 for the BN-only composite. Spectroscopic and morphological evidence confirmed the chemical bonding between BN and SiC, which, together with inter-particle hydrogen bonding among SiC domains, reduces phonon scattering. Furthermore, device-level validation under operational heating conditions demonstrated that the BN–SiC composite TIM effectively suppresses thermal accumulation and enables rapid and uniform heat spreading. In conclusion, the BN–SiC strategy provides a scalable and compositionally tunable design framework for high-performance, thermally isotropic TIMs. Beyond LED packaging, these composites are expected to find applications in advanced power modules, high-frequency devices, and next-generation energy storage systems that require reliable heat dissipation. For future research, it is important to further evaluate mechanical properties at high filler loadings and to explore various polymer matrices and alternative filler combinations to expand the practical applicability and implementation scope of this strategy.

## Figures and Tables

**Table 1:** Optimization of BN:SiC composition ratio in hybrid fillers for balanced vertical and lateral thermal pathways in composite.

Filler Volume %	BN: SiC Weight Ratio	X-Y Plane Thermal Conductivity	Z-Axis Thermal Conductivity
70	1:1	3.32	1.16
	2:1	4.59	1.61
	3:1	4.72	1.42
	4:1	4.85	1.27
	5:1	4.93	1.14
	6:1	5.12	1.09

## Data Availability

The original contributions presented in this study are included in the article. Further inquiries can be directed to the corresponding author.
